# Impaired Removal of the Damaged Mitochondria in the Metabolic Memory Phenomenon Associated with Continued Progression of Diabetic Retinopathy

**DOI:** 10.1007/s12035-023-03534-1

**Published:** 2023-08-18

**Authors:** Renu A. Kowluru, Ghulam Mohammad, Jay Kumar

**Affiliations:** Ophthalmology, Visual and Anatomical Sciences, Wayne State University, Detroit, MI USA

**Keywords:** Diabetes, Metabolic memory, Mitochondria, Mitophagy, Retinopathy

## Abstract

Retinopathy fails to halt even after diabetic patients in poor glycemic control try to institute tight glycemic control, suggesting a “metabolic memory” phenomenon, and the experimental models have demonstrated that mitochondria continue to be damaged/dysfunctional, fueling into the vicious cycle of free radicals. Our aim was to investigate the role of removal of the damaged mitochondria in the metabolic memory. Using human retinal endothelial cells (HRECs), incubated in 20 mM D-glucose for 4 days, followed by 5 mM D-glucose for 4 additional days, mitochondrial turnover, formation of mitophagosome, and mitophagy flux were evaluated. Mitophagy was confirmed in a rat model of metabolic memory where the rats were kept in poor glycemic control (blood glucose ~ 400 mg/dl) for 3 months soon after induction of streptozotocin-induced diabetes, followed by 3 additional months of good control (BG < 150 mg/dl). Reversal of high glucose by normal glucose had no effect on mitochondrial turnover and mitophagosome formation, and mitophagy flux remained compromised. Similarly, 3 months of good glycemic control in rats, which had followed 3 months of poor glycemic control, had no effect on mitophagy flux. Thus, poor turnover/removal of the damaged mitochondria, initiated during poor glycemic control, does not benefit from the termination of hyperglycemic insult, and the damaged mitochondria continue to produce free radicals, suggesting the importance of mitophagy in the metabolic memory phenomenon associated with the continued progression of diabetic retinopathy.

## Introduction

Retinopathy remains one of the most feared complications of diabetes, and despite many technical advances in the field, its pathogenesis remains poorly understood. Hyperglycemia is considered as the main instigator in the development of diabetic retinopathy, but, landmark Diabetes Control and Complications Trial (DCCT), and the follow-up Epidemiology of Diabetes Interventions and Complications (EDIC) studies, have clearly demonstrated that the termination of hyperglycemia does not stop its progression, and retinopathy continues to progress in diabetic patients even after maintaining good glycemic control. Furthermore, if the intensive glycemic control is initiated in the early stages of diabetes, the benefits of good glycemic control last beyond its institution, suggesting a “metabolic memory” phenomenon [1–3]. This metabolic memory phenomenon is successfully duplicated in the experimental models of diabetic retinopathy including in isolated retinal cells in culture and chemically induced diabetic rats or dogs [4–7]. However, the molecular mechanism of the failure of retinopathy to halt progression after removal of the hyperglycemic insult remains obscure.

Reactive oxygen species (ROS) are increased in diabetes, and oxidative stress is considered to play a major role in the pathogenesis of diabetic microvascular complications including retinopathy [8]. Mitochondria, the major cellular source of free radicals, are damaged in diabetes, and retinal mitochondrial structural, functional, and genomic stability is impaired [9, 10]. Mitochondria undergo constant fission and fusion, and these dynamic properties are critical for their optimal function in energy generation. Fusion dilutes the contents of damaged mitochondria by integrating the contents of two mitochondria, and fission segregates the damaged mitochondrial components. Failure of these quality control mechanisms leaves mitochondria as terminally damaged, which threatens the cell-survival [11]. Disrupted mitochondrial dynamics, in addition to negatively impacting mitochondrial function, also results in the buildup of dysfunctional and damaged mitochondria [12]. In diabetic retinopathy, mitochondrial dynamic is imbalanced, mtDNA is damaged, mitochondrial biogenesis is inhibited and mtDNA copy numbers are reduced [10, 13, 14]. Furthermore, experimental models (in vitro and in vivo) have shown that reversal of hyperglycemia by normal glycemia fails to provide any benefit to mitochondrial structural, functional and genomic stability; mitochondria remain fragmented and the damaged mitochondria continue to fuel into the vicious cycle of free radicals [6, 7, 15].

 The damaged/dysfunctional mitochondria can be degraded either by a ubiquitin–proteasome system, which eliminates mitochondrial outer membrane proteins, or by an autophagy-lysosome pathway, “mitophagy.” Mitophagy selectively excludes damaged mitochondria as a whole organelle via a specific autophagic pathway, where damaged mitochondria are engulfed in the double membrane intracellular organelles — “mitophagosomes” for lysosomal degradation [16–18]. The role of mitophagy in the continued progression of diabetic retinopathy is not clear.

The aim of this study was to investigate the role of mitochondrial turnover, and the removal of the damaged mitochondria, in the metabolic memory phenomenon. Using an in vitro model (human retinal endothelial cells, HRECs) of metabolic memory, mitochondrial turnover, mitophagosome formation, and mitophagy flux were evaluated. Our previous studies have shown that in the pathogenesis of diabetic retinopathy, activation of cytosolic matrix metalloproteinases (MMPs) in the retina damages the mitochondria [19]; the effect of inhibition of MMP-9 activation on the removal of the damaged mitochondria was evaluated. Effect of termination of hyperglycemia on mitophagy was confirmed in a rat model of metabolic memory.

## Methods

### Retinal Endothelial Cells

Retinal endothelial cells, isolated from nondiabetic human retina (HRECs, Cell system, Kirkland, WA), from 6 to 8th passage were incubated in 5 mM D-glucose (NG) or 20 mM D-glucose (HG) in DMEM containing 1% fetal calf serum, 9% Nu-serum, and 1 µg/mL endothelial growth supplement for 4 days. High glucose exposed cells either remained in continuous high glucose for 8 days, in the absence or presence of 4 nM MMP-9 inhibitor 1 [20] (Cat no. CAS 1177749–58-4; Sigma-Aldrich, St. Louis, MO, HG and HG/Inh groups, respectively), or after 4 days they were incubated in 5 mM D-glucose for four additional days in a medium supplemented without or with MMP-9 inhibitor (HG-NG and HG-NG/Inh groups, respectively). As an osmotic/metabolic control, each experiment had parallel incubation where HRECs were incubated in 20 mM L-glucose (L-G), instead of 20 mM D-glucose [15, 21]

### Rats

Rats, male, Wistar (~ 200 g BW), were made diabetic by streptozotocin (55 mg/kg body weight), and were allowed to remain either in poor glycemic control (blood glucose > 400 mg/dL; PC group) or in good glycemic control (blood glucose > 150 mg/dL; GC group) for 6 months. A group of rats were maintained in poor glycemic control for 3 months, which was followed by good glycemic control for three additional months (PC-GC). The age-matched normal rats were used as controls. The rats in the PC group received 1–2 IU insulin 4 to 5 times a week, and those in GC received insulin twice daily (5–7 IU total). Rats were weighed two times a week; their blood glucose was measured once every week; these methods are used routinely in our laboratory [15, 21].

### Mitochondrial Turnover

Mitochondrial turnover was determined using MitoTimer, the assay based on the principle that the incorporation of pMitoTimer, a fluorescent timer reporter (DsRed1-E5-reporter) fluoresces green in the newly synthesized mitochondria, but shifts irreversibly to red spectrum over time [22]. pMitoTimer plasmids, obtained as transformed bacteria in stab culture (Cat. no. 52659; Addgene, Watertown, MA), were streaked onto the LB-agar (MP Biomedicals, Irvine, CA) plates supplemented with 50 μg/ml Kanamycin (Sigma-Aldrich). Plates were incubated overnight at 37 °C, and the colonies were picked the following day and incubated overnight at 37 °C with 5 ml LB medium (supplemented with Kanamycin, 50 μg/ml). Plasmid DNA was isolated and purified using a GenElute Plasmid Miniprep Kit (Cat. no. PLN70, Sigma-Aldrich) according to the manufacturer’s instructions. A group of cells from 5 to 7th passage were washed with the Opti-MEM transfection medium (Cat. No, 31,985,062, Thermo Fisher Scientific, Waltham, MA), and incubated with the transfection complex containing 500 ng pMitoTimer plasmid DNA in the transfection reagent (lipofectamine 3000 with P3000 reagent, Cat. No. L3000008; Thermo Fisher Scientific) for 8 h at 37 °C. Cells were then washed with DMEM (2 ×), and MitoTimer expression was induced by the addition of 0.5 µg/ml doxycycline. After 4 h of incubation, the cells were incubated as per the experimental conditions, and imaged under a Zeiss Apotome (Carles Zeiss, Inc., Chicago, IL) using a 20 × objective at 550 nm excitation and 620 nm wavelengths. The ratio of the fluorescence intensity in the red and green channels was calculated using the Zeiss software module [23]. As a positive control, cells incubated with 25 µM chloroquine were also included in each experiment [24].

### Autophagic Vacuoles

Autophagic vacuole formation was assessed in live cells by an autophagy detection kit (cat. no. ab139484; Abcam, Cambridge, MA), using a cationic amphiphilic tracer (CAT) dye. Briefly, at the termination of the experimental incubations, cells were washed with the assay buffer, and incubated in the dark with the green detection reagent for 30 min at room temperature. Cells were then mounted using DAPI-containing (blue) Vectashield mounting medium (Vector Laboratories) to counterstain the nuclei, and imaged under a Zeiss Apotome at 20 × objective. The mean intensity was determined using the Zeiss software module. Each experiment included rapamycin (1 µM) as an autophagy inducer control [25].

Autophagic vacuole formation was also confirmed by quantifying fluorescence in a microplate reader using the same autophagy detection kit, as used for imaging. Cells were incubated with the detection reagent at 37 °C for 30 min. After washing with the assay buffer to remove the excess dye, 100 μl of the assay buffer was added, and the fluorescence was measured at 480 nm excitation and 530 nm emission wavelengths [26]. Relative fluorescence intensity (arbitrary units) was calculated considering the values obtained from HRECs in normal glucose as 100%.

### Mitophagosome

Mitophagosome formation was estimated by the colocalizing mitochondria with lysosomes, a technique which takes the advantage that lysosomes degrade the autophagosomes bound to the damaged mitochondria [27]. LysoTraker (LTR, red), an acidotropic fluorescent probe, which labels and tracks acidic organelles in the living cells [28], was used to label lysosomes, and MiroTraker green (MTR) to label the mitochondria. In brief, after experimental incubations, HRECs were incubated for 30 min at 37 °C with 100 nM LTR (Cat. No. L7528, Thermo Fisher Scientific) and 200 nM MTR (Cat. No. M7514, Thermo Fisher Scientific). The coverslips were washed with PBS, and imaged in a Zeiss Apotome under a 20 × objective [27]. The images were obtained from 6 to 8 cells/group, and three different cell preparations were imaged. The intensities of LTR and MTR were quantified using the Zeiss software module, and Pearson correlation coefficient between LTR and MTR was plotted.

### Mitophagy

Mitophagy was quantified using a mitophagy detection kit (Cat. No. MD0110; Dojindo Molecular Technologies, Rockville, MD). After experimental incubations, HRECs were washed with DMEM, and incubated with 100 nM Mtphagy Dye and 1 µM Lyso Dye for 30 min at 37 °C. Cells were then washed with PBS (2 ×), and imaged under a Zeiss Apotome using a 20 × objective [29]. Cells incubated in 25 mM carbonyl cyanide m-chlorophenylhydrazone (CCCP; Cat no,C2759; Sigma-Aldrich) were used as a positive control.

In rat retina, mitophagy was performed by flow cytometry technique using MitoTracker deep red (Cat No. M22426, Thermo Fisher Scientific) [30]. Briefly, freshly isolated retina was cut into small pieces, and incubated with 50 µl of Accumax™ (Cat No. A7089, Sigma-Aldrich) for 10 min at 37 °C. Digested retinal tissue was washed with DMEM containing 10% fetal bovine serum, and filtered through a 40-μm cell strainer [31]. Cells were then incubated with 100 nM MitoTracker deep red for 30 min at 37 °C, and after washing cells (3 ×) with the flow buffer (0.5% BSA in PBS), they were scanned under FL3 640 nm wavelength in a BD Accuri C6 plus flow cytometer (BD Biosciences, San Jose, CA, USA). Raw flow cytometry standard files were analyzed by the FlowJo v10.8.1 software.

### Statistical Analysis

GraphPad Prism (GraphPad Software, San Diego, CA, USA) was used for statistical analysis, and significance of variance was calculated by one-way ANOVA; *p* value < 0.05 was considered as statistically significant.

## Results

**Fig. 1:**
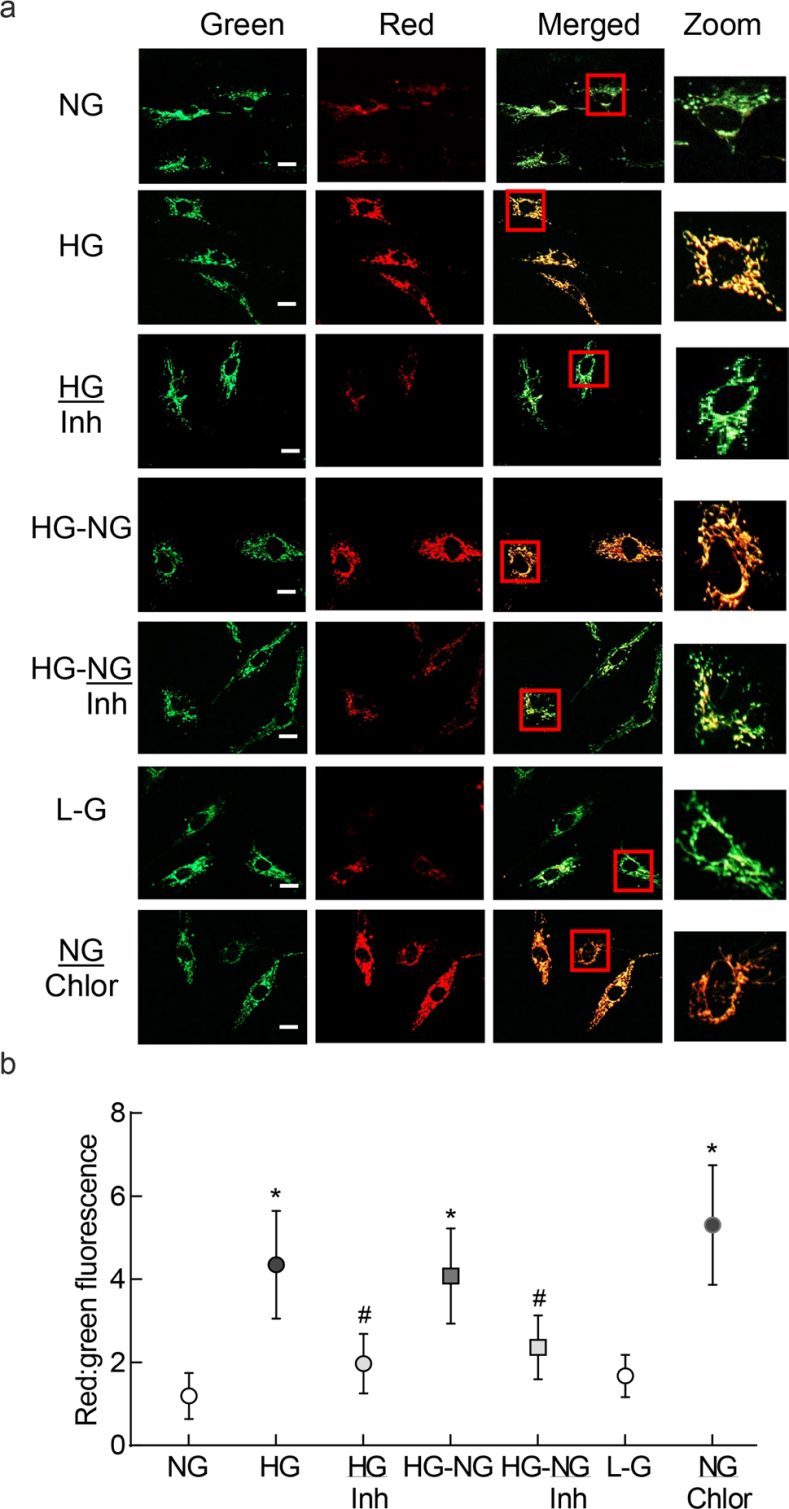
Effect of reversal of high glucose on mitochondrial turnover in retinal endothelial cells. Mitochondrial turnover was determined by transfecting HRECs with MitoTimer, and inducing its expression by doxycycline. **a** Cells were imaged in a Zeiss Apotome using a 20 × objective (scale bar = 20 µm). **b** Ratio of the red and green fluorescence was plotted. Data are represented as mean ± SD of the values obtained from three cell preparations, and imaging of 5–6 cells/preparation. NG = 5 mM D-glucose; HG and HG/Inh = 20 mM D-glucose, without or with MMP-9 inhibitor, respectively; HG-NG and HG-NG/Inh = 4 days of 20 mM D glucose, followed by 4 days of 5 mM D-glucose, without or with MMP-9 inhibitor, respectively; L-G = 20 mM L-glucose; NG/Chlor = 5 mM D-glucose + chloroquine. * and # *p* < 0.05 compared to NG or HG, respectively

Mitochondria are damaged in hyperglycemic milieu, and their biogenesis and dynamics are impaired [10, 14]; to determine the effect of high glucose insult on the mitochondrial turnover, MitoTimer fluorescent probe was employed. Compared to normal glucose, cells in high glucose had shifting of the green fluorescence to red, and the ratio of red to green fluorescence was significantly higher, suggesting increased accumulation of the damaged mitochondria. Since MMP-9 plays a major role in mitochondrial damage in diabetes [19], the effect of inhibition of MMP-9 on mitochondrial turnover was determined. Supplementation of high glucose medium with a specific inhibitor of MMP-9 (HG/Inh group) ameliorated mitochondrial turnover, and red-green fluorescence intensity ratio was significantly decreased, compared to cells in high glucose alone. Removal of high glucose insult fails to protect mitochondria from glucose-induced damage [6, 7], the effect of reversal of high glucose with normal glucose on mitochondrial turnover was determined. Four days of normal glucose, which had followed 4 days of high glucose (HG-NG group), had no effect on the mitochondrial turnover, and red-green fluorescence signal ratios were similar in HG and HG-NG groups, suggesting that the damaged mitochondria continued to accumulate even when high glucose insult was terminated. However, addition of MMP-9 inhibitor during the 4 days of normal glucose, that had followed four days of high glucose exposure (HG-NG/Inh group), ameliorated mitochondrial turnover, and the values were significantly different than those in HG and HG-NG groups (*p* < 0.05). Cells in 20 mM L-glucose had similar red-green fluorescence ratio as those in 5 mM D-glucose (Fig. 1a and b).

**Fig. 2:**
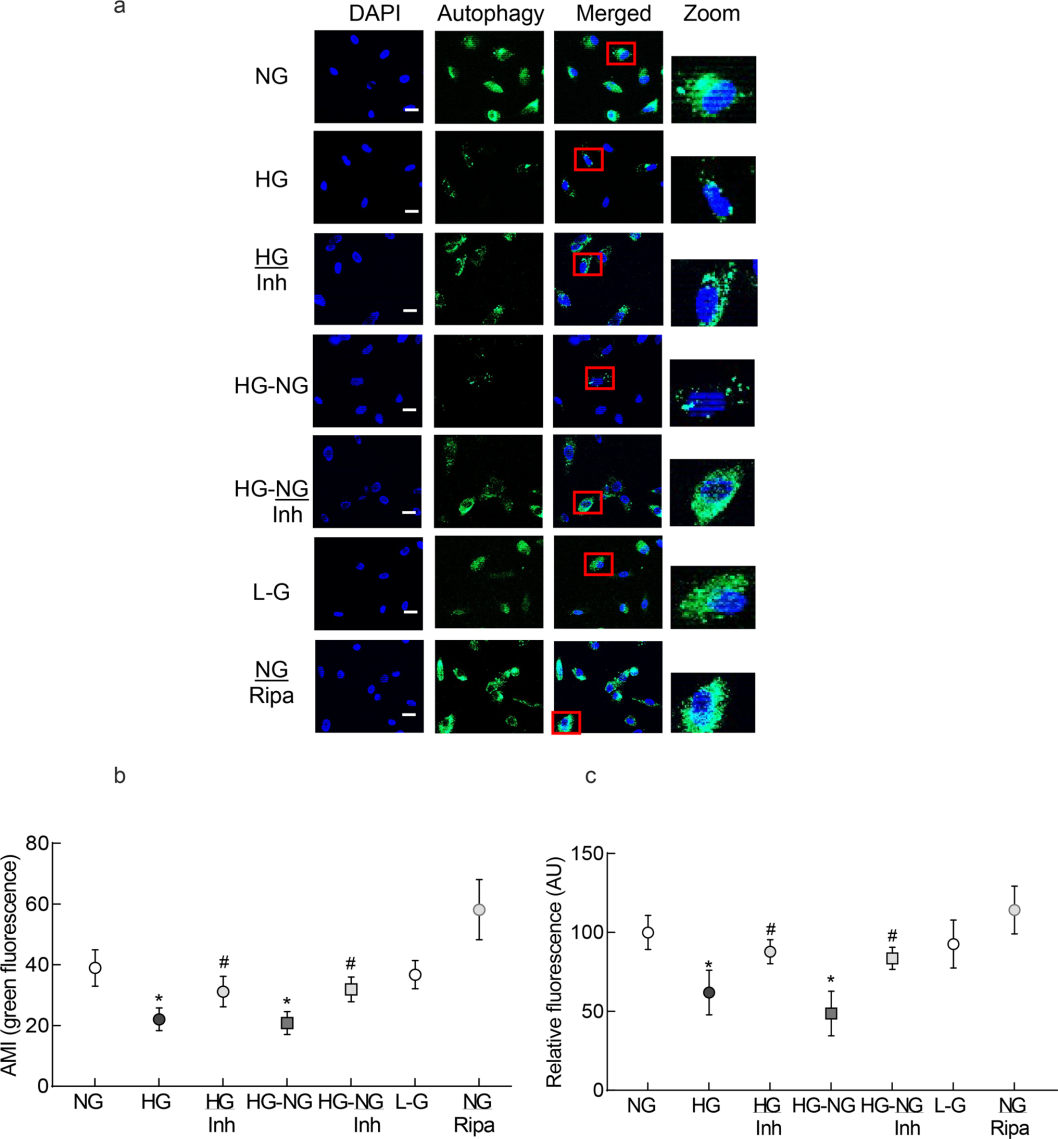
Autophagosome formation and reversal of high glucose insult. Autophagosomes were quantified in live cells using a cationic amphiphilic tracer to selectively label autophagic vacuole. **a** Cells mounted in DAPI-containing (blue) Vectashield mounting medium were imaged under a Zeiss Apotome at 20 × objective (scale bar = 20 µm). **b** Mean intensity of green fluorescence was plotted. Values are mean ± SD obtained from 3 to 4 cell preparations, with measurement made in 6 or more cells/preparation. **c** Plot of relative fluorescence intensity at 480 nm excitation and 530 nm emission wavelengths in arbitrary units (AU), considering values from normal glucose as 100%. NG and HG = 5 mM and 20 mM D-glucose; HG/Inh = 20 mM D-glucose or with MMP-9 inhibitor; HG-NG and HG-NG/Inh = 20 mM D glucose, followed by 5 mM D glucose, without or with MMP-9 inhibitor, respectively; L-G = 20 mM L-glucose; NG/Ripa = 5 mM D-glucose + Ripamycin. * and # *p* < 0.05 vs. NG or HG, respectively

Damaged mitochondria are engulfed in the autophagosomes for their lysosomal degradation [17]; the effect of high glucose on autophagosome formation was investigated. Consistent with mitochondrial turnover, compared to normal glucose, high glucose decreased the number of the fluorescent autophagic vacuoles, as indicated by decreased autophagy dye staining (Fig. 2a and b), and MMP-9 inhibitor prevented decrease in the autophagosome formation (*p* > 0.05 vs NG). Termination of high glucose insult, in addition to preventing decrease in mitochondrial turnover, also failed to prevent decrease in autophagosomes. However, supplementing normal glucose, which had followed high glucose, with an MMP-9 inhibitor (HG-NG/Inh group), prevented decrease in the autophagosome formation, and the values in HG-NG/Inh group were significantly different from those in HG or HG-NG groups. L-glucose (20 mM) had no effect on the number of autophagosomes (Fig. 2a and b). Consistent with the immunofluorescence results, quantitative assay also showed a significant decrease in relative fluorescence intensity in high glucose, compared to normal glucose. Four days of normal glucose, after 4 days of high glucose (HG-NG group), had no beneficial effect on autophagosome formation; the values in HG and HG-NG groups were not different from each other. However, MMP-9 inhibitor addition during normal glucose incubation (HG-NG/Inh group) ameliorated decrease in autophagosome formation, and the values in NG, HG/Inh, and HG-NG/Inh were not different from each other (*p* > 0.05; Fig. 2c).

**Fig. 3:**
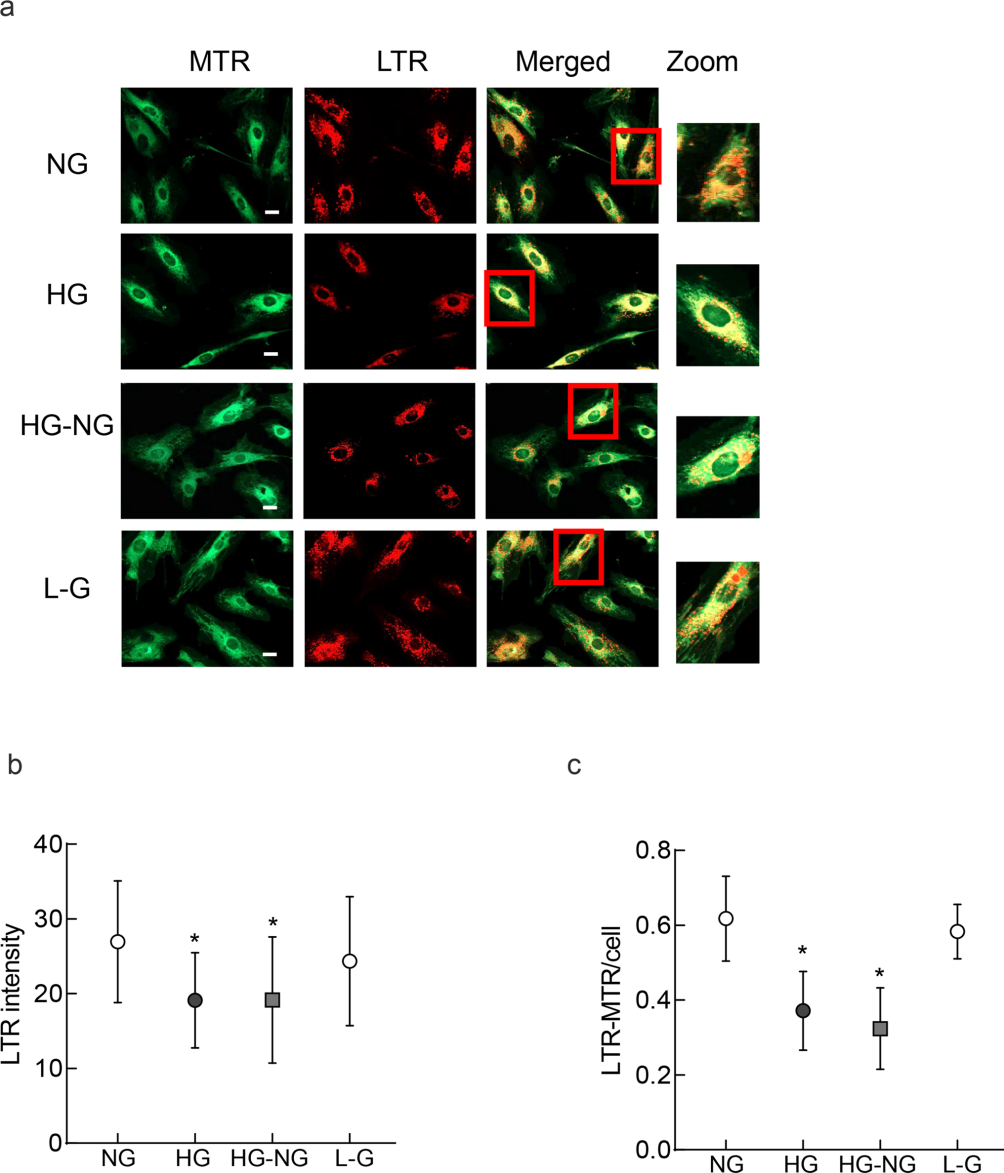
Reversal of high glucose and mitophagosome formation. Lysosomes labeled with an acidotropic fluorescent probe LysoTracker (LTR, red) and mitochondria with MiroTraker (MTR, green) were **a** imaged using a 20 × objective (scale bar = 20 µm). **b** Mean intensity of LTR and **c** the ratio of LTR and MTR intensities were plotted. Each experiment was repeated in 3 different cell preparations with 6–8 cells imaged/group/preparation

Lysosomes clear the damaged mitochondria engulfed in the autophagosomes [16, 17]; to investigate the effect of high glucose on the clearance of the damaged mitochondria, mitophagosome formation was determined by staining lysosomes with LTR and mitochondria with MTR. Lysosome staining was reduced significantly in the cells in high glucose, compared to normal glucose, and remained subnormal in HG-NG group (Fig. 3a and b). Consistent with the staining, Pearson correlation coefficient between LTR and MTR was also significantly less in HG and HG-NG groups, compared to cells in normal glucose (*p* < 0.05; Fig. 3c). Cells in 20 mM L-glucose and 5 mM D-glucose had similar LTR intensity and LTR-MTR Pearson correlation coefficient.

**Fig. 4:**
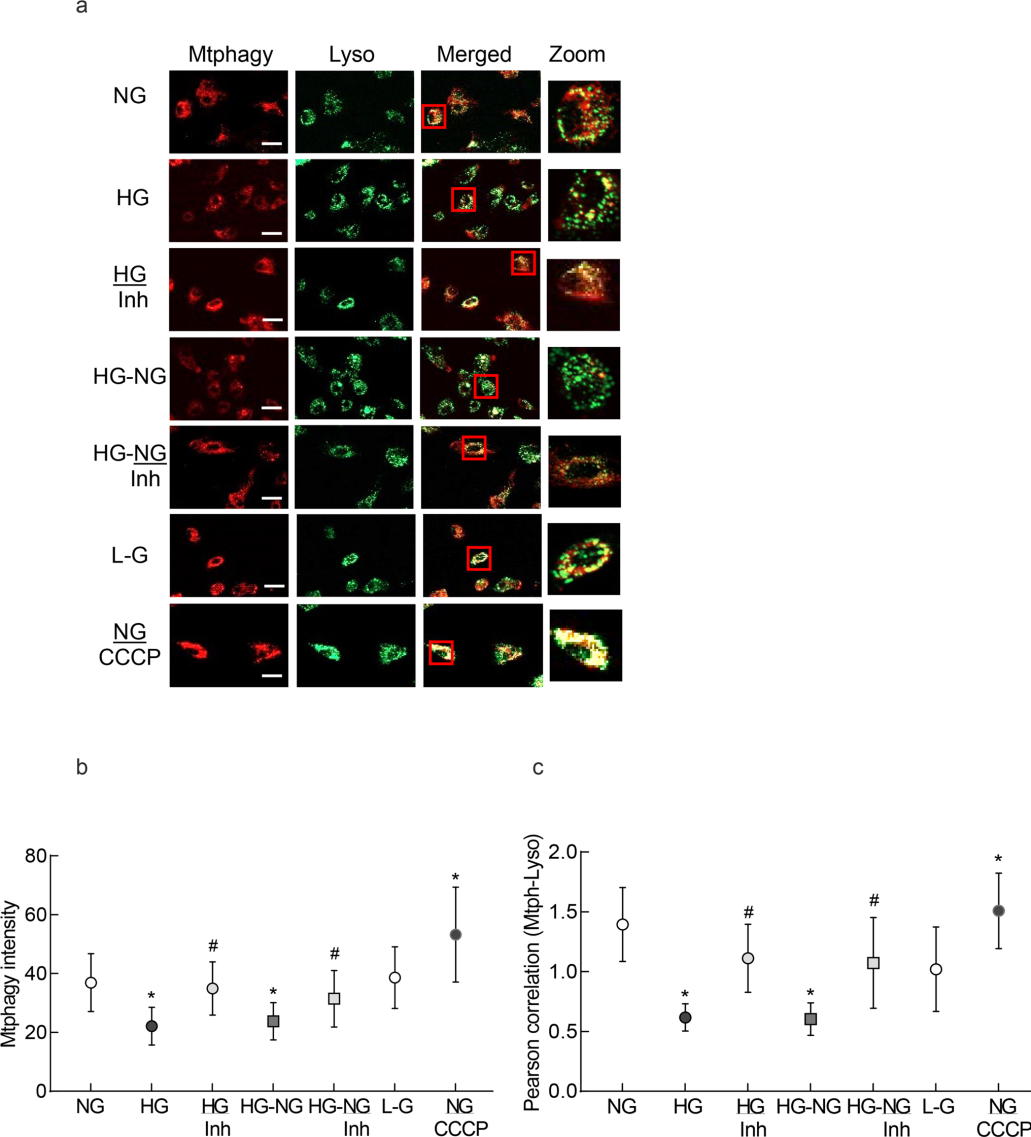
Effect of reversal of high glucose on mitophagy flux. Retinal endothelial cells stained with Mtphagy Dye and Lyso Dye were **a** imaged using a 20 × objective (scale bar = 20 µm). **b** Arithmetic mean intensity of Mtphagy dye and **c** Pearson correlation coefficient between Mtphagy and Lyso dye were plotted. Values are mean ± SD obtained from 3 to 4 cell preparations, with each measurement made in duplicate. NG = 5 mM D-glucose; HG and HG/Inh = 20 mM D-glucose, without or with MMP-9 inhibitor, respectively; HG-NG and HG-NG/Inh = 4 days of 20 mM D glucose and 4 days of 5 mM D-glucose, without or with MMP-9 inhibitor, respectively; L-G = 20 mM L-glucose; NG/CCCP = 5 mM D-glucose + carbonyl cyanide m-chlorophenylhydrazone. * and # *p* < 0.05 vs. NG or HG, respectively

In accordance with the mitophagosome formation, cells in high glucose, compared to normal glucose, had decreased mitophagy flux, as indicated by decreased Mtphagy dye staining (Fig. 4a), and the intensity of Mtphagy dye and Pearson correlation between Mtphagy and Lyso- dyes were significantly less in the HG group compared to the NG group (Figs. 4b and c). Reversal of high glucose with normal glucose (HG-NG group) failed to prevent decrease in mitophagy flux. Supplementation with MMP-9 inhibitor in high glucose (HG/Inh group), or during normal glucose exposure, which had followed high glucose (HG-NG/Inh group), ameliorated decrease in mitophagy. Values in HG and HG-NG groups were similar (*p* > 0.05), but were significantly different from those in the HG/Inh or HG-NG/Inh groups (*p* < 0.05). Cells in 20 mM L-glucose or in 5 mM D-glucose were not different from each other.

### Rats

Rats in continuous poor glycemic control for 6 months (PC group), as reported previously [15, 21], had significantly lower body weight (~ 320 g), and higher blood glucose (~ 400 mg/dl) and urine output (> 120 ml/24 h), compared to the rats in the Norm or GC groups (~ 520 g, ~ 110 mg/dl, and < 15 ml/24 h, respectively). During the 3 months of poor glycemic control, rats in the PC-GC group and the PC group had similar body weight, blood glucose, and urine output, and after 3 months of good glycemia in these rats, the values became similar to those in the Norm or GC groups. There was no significant difference between these metabolic parameters in the rats in the Norm and GC groups (*p* > 0.05).

**Fig. 5:**
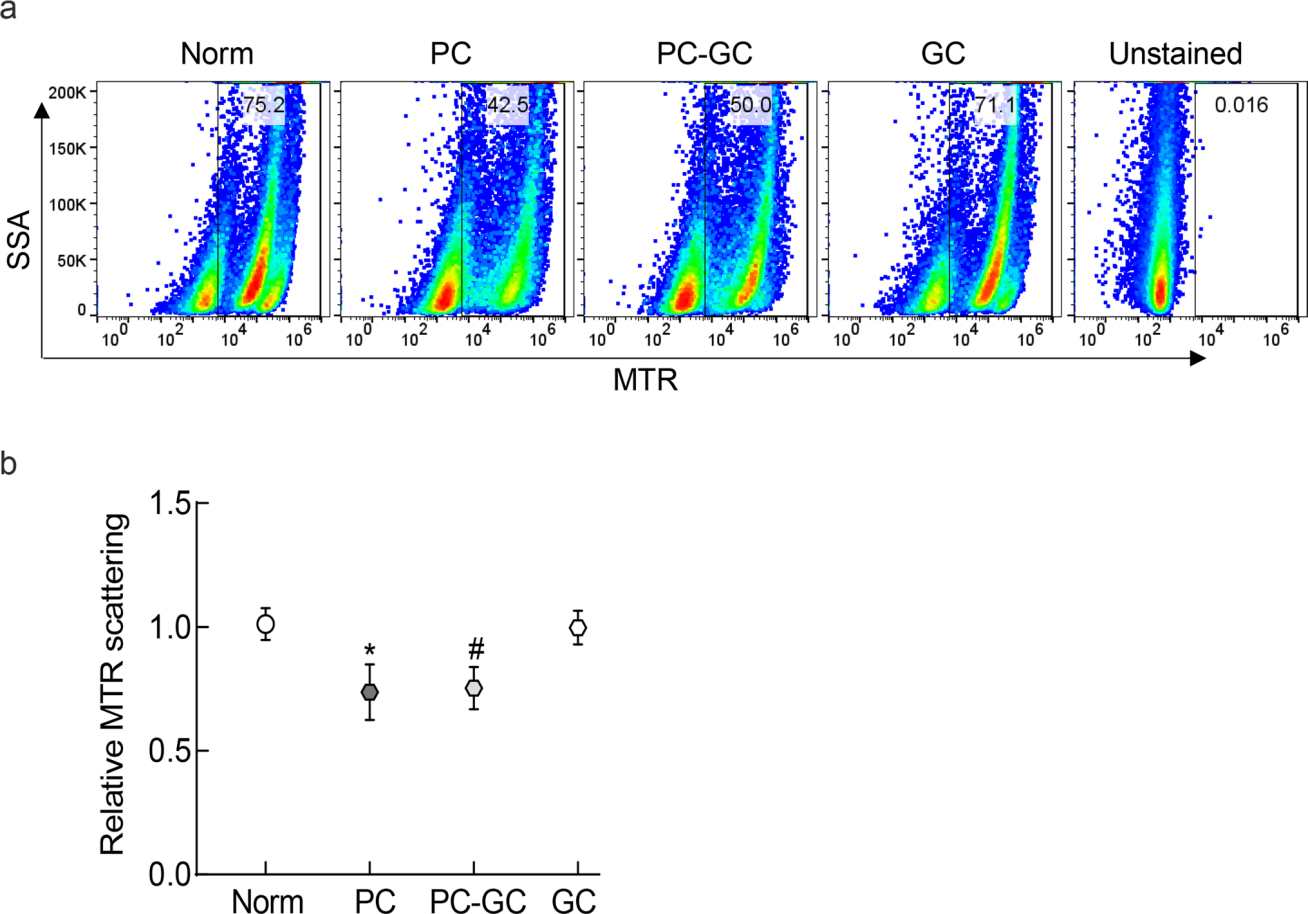
Effect of re-institution of normoglycemic control in diabetic rats on mitophagy. In retinal cell preparation, **a** mitophagy was determined by flow cytometry technique using MitoTracker deep red. Raw flow cytometry standard files were analyzed by the FlowJo v10.8.1 software, and **b** the relative fold change in the scattering was plotted (SSA = side scatter A). Values are represented as mean ± SD obtained from 5 to 7 rats in each group. Norm = normal; PC = rats in continuous poor glycemic control for six months; PC-GC = rats in poor glycemic control for three months, followed by 3 months of good glycemic control; GC = rats in continuous good glycemic control for 6 months. *p < 0.05 compared to normal or GC, #*p* > 0.05 vs PC

Analysis of mitophagy using flow cytometry showed significantly reduced scattering of MitoTracker deep red in the retina from diabetic rats compared to normal rats, and reversal of poor glycemia with good glycemia (PC-GC group) failed to provide any benefit; the values in the PC and PC-GC groups were similar to each other (*p* > 0.05), but were significantly different from those in normal group. Institution of good glycemic control soon after induction of diabetes (GC group) had values that were not different from those obtained from normal rats (*p* > 0.05; Fig. 5a and b).

## Discussion

Mitochondria become dysfunctional in diabetes, and their DNA is damaged, and in the pathogenesis of diabetic retinopathy, mitochondrial damage accelerates capillary cell apoptosis, a phenomenon which precedes the development of histopathology characteristic of diabetic retinopathy [32]. Due to damage to the mtDNA, transcription of mtDNA-encoded genes is reduced, and the electron transport chain system is compromised, fueling into a self-propagating vicious cycle of free radicals, and the retinopathy continues to develop [9, 10, 13]. Mitochondrial quality control is critical for cellular integrity, and since damaged mitochondria continue to produce ROS, clearance of these damaged/dysfunctional mitochondria is important to maintain functional mitochondrial population. Here, our exciting results show that the clearance of the damaged mitochondria remains subnormal even after removal of the hyperglycemic insult; turnover of the mitochondria remains suboptimal, and the mitophagy process continues to be impaired with reduction in mitophagosome formation. However, preventing mitochondrial damage by inhibiting MMP-9 activation, an enzyme implicated in mitochondrial damage [19], during the reversal phase, ameliorates decrease in mitochondrial turnover, and improves the mitophagy process. The results are confirmed in a rat model showing that the mitophagy remains compromised even after 3 months of good glycemic control, which has followed 3 months of poor glycemic control, clearly demonstrating that the poor turnover/removal of the damaged mitochondria, initiated during the poor glycemic control, does not benefit from the termination of hyperglycemic insult, and suggest its role in the metabolic memory phenomenon associated with the continued progression of diabetic retinopathy.

Mitochondrial quality control is regulated by two opposing processes, removal of the damaged organelles or their components, and mitochondrial biogenesis forming more mitochondria. Mitochondrial turnover, which reflects the total sum of degradation versus mitochondrial biogenesis, is critical for cellular homeostasis as the damaged mitochondria continues to produce less ATP and more ROS [22, 33, 34]. Our previous work has demonstrated that retinal mitochondrial biogenesis is impaired in diabetes, and it continues to be impaired even after termination of hyperglycemic insult, contributing in the metabolic memory phenomenon [35]. Here, we show that high glucose also impairs mitochondrial turnover, and the turnover remains suboptimal even when high glucose insult is replaced by normal glucose. In support, higher levels of retinal mitochondria primed for degradation are observed in genetically manipulated mouse model of diabetes [36], and the vicious cycle of free radicals does not benefit from the termination of high glucose insult [7, 10, 37].

Damaged mitochondria can either be degraded via mitophagy or rescued by mitochondrial dynamics [38]. Mitochondria are very dynamic, which is important in segregating their functional and damaged elements [39], and in diabetic retinopathy, retinal mitochondrial dynamics is imbalanced with increased fission and decreased fusion. Furthermore, reversal of hyperglycemic insult does not benefit the fission–fusion process, and mitochondria remain fragmented [15, 21]. Mitophagy, a process where the damaged mitochondria are engulfed in double membrane autophagosomes “mitophagosomes” for their lysosomal degradation [16–18]. Under normal physiological conditions, mitophagy has an essential role in the basal mitochondrial turnover and maintenance, and can be stimulated by a variety of pathological stimuli [40, 41]. While impaired or insufficient mitophagy results in the accumulation of poorly functional/damaged mitochondria with suboptimal ATP production and high levels of ROS, increased mitophagy results in decreased mitochondrial numbers [42, 43]. Defective mitophagy is reported in pathogenesis of many diseases, in particular to age-related sporadic disorders, such as Parkinson’s disease, Alzheimer’s disease, and cardiomyopathy [44–46]. As mentioned above, retinal mitochondrial are damaged in diabetes, their membrane potential is reduced, and dynamics is imbalanced, and mitochondrial damage does not get a break from the termination of hyperglycemia [9, 10, 13]. Here, we show that high glucose also attenuates formation of mitophagosome and reduces removal of the damaged mitochondria, and reversal of high glucose with normal glucose fails to provide any benefit to the removal of the damaged mitochondria, further contributing in the continuous accumulation of the damaged mitochondria.

Activation of MMPs in diabetic retinopathy is implicated in mitochondrial damage, and inhibition of MMP-9 prevents mitochondrial damage and the development of retinopathy in diabetic mice [19, 47]. Our results here demonstrate that the direct inhibition of mitochondrial damage by MMP-9 inhibitor during the normal glucose exposure, which has followed high glucose exposure, restores mitochondrial turnover and the mitophagy process, further supporting the role of mitophagy in the metabolic memory associated with the continued progression of diabetic retinopathy. In support, direct regulation of mitochondrial damage by lipoic acid in a rat metabolic memory model during the good glycemic control, which has followed poor control, restores mitochondrial biogenesis. Moreover, supplementation of a Dnmt inhibitor during the good glycemic control prevents downregulation of Mitofusin 2, a mitochondrial fusion protein, by preventing DNA hypermethylation of its promoter. In the same experimental model of metabolic memory, these therapies also cease progression of diabetic retinopathy [6, 35].

Similar results from an animal model of metabolic memory, where mitochondrial function and biogenesis remain subnormal even after re-institution of normal glycemia, after a period of hyperglycemia [35], showing continued impaired mitophagy process further confirms the role of mitophagy in the metabolic memory phenomenon. Institution of early intensive glycemic control in diabetic patients with poor glycemic control reduces further progression of retinopathy, and even after decades, former intensive therapy participants in DCCT have better visual function compared to those in former conventional group [48]. The same phenomenon is also duplicated in animal models; good glycemic control instituted soon after diabetes protect mitochondrial dysfunction and fragmentation, and continued development of diabetic retinopathy [5, 21, 35]. Here, we show that rats in GC group have similar mitophagy as observed in normal rats, further supporting the importance early, and continued, good glycemic control for a diabetic patient.

We recognize that our study is focused on the mitophagy process in metabolic memory, but we cannot rule out the role of other pathways associated with the removal of the damaged mitochondria including degradation of the damaged outer mitochondrial membrane proteins by the proteasome, or fusion of mitochondria-derived vesicles with lysosomes to degrade oxidized mitochondrial proteins [49, 50]; their role in the progression of diabetic retinopathy remains to be investigated.

**Fig. 6:**
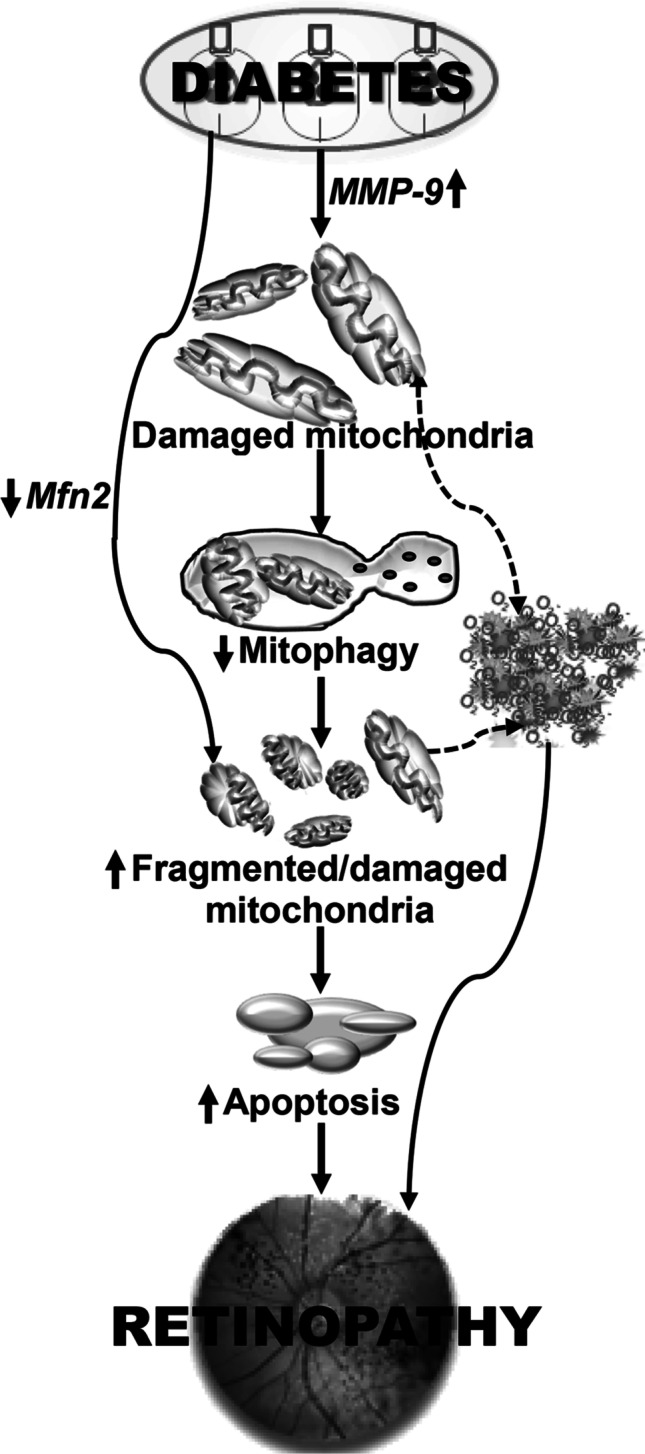
Working model. Diabetes activates MMP-9 and damages the mitochondria, and impaired mitophagy process fails to clear the damaged mitochondria. Damaged mitochondria accelerate capillary cell apoptosis, resulting in the development of retinopathy. In addition, mitochondrial dynamics is also imbalanced, and inhibition of mitochondrial fusion protein, mitofusin 2 (Mfn2), leads to further accumulation of the fragmented mitochondria, resulting in continued accumulation of ROS. Termination of hyperglycemic insult does not give any break, and the ROS production by the damaged/fragmented mitochondria continue to accelerate cell death, failing to provide any benefit to the progression of diabetic retinopathy

In conclusion, results from experimental models have demonstrated an important role of mitophagy in the metabolic memory phenomenon associated with the continued progression of diabetic retinopathy. Due to poor turnover and the removal of the damaged mitochondria, the dysfunctional mitochondria continue to accumulate, the electron chain system remains compromised and ROS productions continues. Removal of high glucose insult does not help with the mitophagy process, and poor-quality mitochondria continue to produce ROS (Fig. 6). Furthermore, maintenance of early, and sustained, good glycemic control prevents accumulation of the damaged mitochondria, strengthening the importance of tight and sustained glycemic control. Directly preventing mitochondrial damage during the good glycemic control, however, improves mitochondrial turnover and facilitates mitophagy, which provides the possible additional therapeutic opportunities for patients to slow down/halt progression of diabetic retinopathy, and prevent their vision loss.

## Data Availability

RAK is the guarantor of this work and, as such, had full access to all the data in the study and takes responsibility for the integrity of the data and the accuracy of the data analysis.
